# Towards Molecular Profiling in Multiple Myeloma: A Literature Review and Early Indications of Its Efficacy for Informing Treatment Strategies

**DOI:** 10.3390/ijms19072087

**Published:** 2018-07-18

**Authors:** Wolfgang Willenbacher, Andreas Seeber, Normann Steiner, Ella Willenbacher, Zoran Gatalica, Jeff Swensen, Jeffery Kimbrough, Semir Vranic

**Affiliations:** 1 Department of Hematology and Oncology, Innsbruck Medical University, 6020 Innsbruck, Austria; wolfgang.willenbacher@tirol-kliniken.at or Wolfgang.Willenbacher@oncotyrol.at (W.W.); andreas.seeber@tirol-kliniken.at (A.S.); Normann.Steiner@i-med.ac.at (N.S.); ella.willenbacher@i-med.ac.at (E.W.); 2 Oncotyrol, Center for Personalized Cancer Medicine, 6020 Innsbruck, Austria; 3 Caris Life Sciences, Phoenix, AZ 85040, USA; zgatalica@carisls.com (Z.G.); jswensen@carisls.com (J.S.); jkimbrough@carisls.com (J.K.); 4 College of Medicine, Qatar University, Doha 2713, Qatar

**Keywords:** plasma cell dyscrasias, multiple myeloma, molecular profiling, treatment

## Abstract

Multiple myeloma (MM), the second most common hematologic malignancy, is characterized by the clonal expansion of plasma cells. Despite dramatic improvements in patients′ survival over the past decade due to advances in therapy exploiting novel molecular targets (immunomodulatory drugs, proteasome inhibitors and monoclonal antibodies), the treatment of relapsed and refractory disease remains challenging. Recent studies confirmed complex, dynamic, and heterogeneous genomic alterations without unifying gene mutations in MM patients. In the current review, we survey recent therapeutic strategies, as well as molecular profiling data on MM, with emphasis on relapsed and refractory cases. A critical appraisal of novel findings and of their potential therapeutic implications will be discussed in detail, along with the author’s own experiences/views.

## 1. Introduction

Multiple myeloma (MM) is the second most common hematologic malignancy (10% of all) and belongs to the spectrum of plasma cell dyscrasias [1,2,3,4]. It encompasses a variety of entities ranging from indolent monoclonal gammopathy of unknown significance (MGUS) and smoldering MM to symptomatic MM or highly aggressive plasma cell leukemias [5,6]. MM is characterized by a clonal proliferation of plasma cells in bone marrow and sometimes extra-medullary tissues, usually with a concomitant excessive production of monoclonal paraproteins (intact immunoglobulins and/or free light chains). 

In the past two decades, significant improvements in the treatment and outcome of MM patients have been made through the induction of novel treatment modalities with proteasome inhibitors (PI), immunodulatory drugs (IMiDs), monoclonal antibodies, and histone deacetylase (HDAC) inhibitors [7,8,9]. 

The introduction of the new treatment principles enhanced the effect of autologous stem cell transplantation, introduced in MM therapy from the 1980’s onwards [10], combining autologous stem cell transplantation (ASCT) with high efficiency modern induction therapies like bortezomib-lenalidomid-dexamethasone (VRd) [11].

Using induction therapies combining PIs and IMiDs, patients can now frequently enter ASCT in already deep responses, resulting in high rates of MRD (minimal residual disease) negative states in a high proportion of patients after consolidating high dose therapy and ASCT. The net benefit of adding ASCT to triplet inductions has been proven in several trials and a recent meta-analysis [12]. On the other hand, this multi-dimensional approach clearly compares favorably to older data on ASCT alone approaches.

Furthermore, MM is a clinically and genetically heterogeneous disease, and treatment remains challenging, particularly in relapsed/refractory MMs [13,14,15]. However, MM is characterized by the presence of two types of subclonal evolutions, including the linear one (acquisition of new mutations over time in the clone), and predominantly, the branching evolution pattern, where subclones diverge with the acquisition of additional mutations [16]. The concept of subclonality in MM is a critically important issue for detecting biomarkers and optimizing therapy [13,14,16].

In light of the above-mentioned issues, we surveyed the recent literature on the molecular characteristics and treatment modalities for patients with MM, with a special emphasis on the refractory/relapsed forms MM (RRMM) setting.

## 2. Genomic Profile of Multiple Myeloma

Multiple myeloma (MM) harbors various chromosomal and genetic alterations. According to its ploidy, MM can be divided into two groups: hyperdiploid and non-hyperdiploid [16,17]. Hyperdiploid MM is characterized by trisomies affecting chromosomes 3, 5, 7, 9, 11, 15, 19, and/or 21 [17,18], while non-hyperdiploid cancers harbor IgH translocations, including among others, t(4;14), t(6;14), t(11;14), t(14;16), and t(14;20). Of note, t(4;14), t(14;16) and t(14;20), along with del(17p) alterations, are considered highest-risk changes (poor prognosis), whereas t(6;14) and t(11;14) carry standard risk [16,19,20]. It appears that these genomic alterations may also predict the response to certain treatment modalities. Thus, MM cases that are positive for t(4;14) may exhibit increased sensitivity to immunomodulatory drugs, proteasome inhibitors, and several targeted drugs [21]. Many other chromosomal aberrations have been described in patients with MM [22,23], and have been incorporated into widely used risk prediction models (e.g., mSMART, available at: https://www.msmart.org/mm-treatment-guidelines.html). Negative prognostic significance has been attributed especially to t(14;20), t(14;16), and 1q gains.

On the subchromosomal level, Lin et al. (2018) using the Illumina TruSight RNA Pan-Cancer Panel (1385 genes total) recently described 10 novel fusions (e.g., *HGF*/*CACNA2D1* and *SMC3*/*MXI1*) in 21 patients with MM [22]. 

With the use of high throughput technologies, such as gene expression profiling technology, molecular classifications of MM have been suggested [16]. The first was proposed by Bergsagel and colleagues [24], and included eight different subgroups, predominantly based on *CCND1* (cyclin D1) gene expression status and 14q32 translocations. Cyclin D1 dysregulations are one of the key molecular events in the pathogenesis of MM [25]. Additional molecular classifications revealed novel molecular targets and biomarkers; however, these classifications have not led to a uniform molecular classification of MM and have not significantly affected the management of the disease. On the other hand, gene expression studies have been optimized to develop highly prognostic molecular tests (e.g., the GEP70 assay) [26].

The mutational profile of MM has also recently been explored in several studies, revealing a complexity of genetic alterations and diversity related to its subclonal evolution (summarized in a systematic review of Weaver and Tariman) [27]. The mutational profile of MM is characterized by both different mutations within the subclones (e.g., *BRAF* mutation in 50%) and multiple mutations within the same signaling pathway (e.g., *KRAS*, *NRAS* and *BRAF* mutations within the MAPK pathway) [14,16,28]. In contrast to some other hematologic malignancies [e.g., hairy cell leukemia (*BRAF*) and Waldenström’s macroglobulinemia (*MYD88*)], MMs do not harbor any specific or unifying mutation(s) [16]. The most common gene alterations in MM have been reported to be in the *KRAS* (20%), *NRAS* (20%), nuclear factor-κB (17%), *TP53*, *BRAF*, *FAM46C*, *DIS3*, *ATM*, and *CCND1* genes (approximately 10%, respectively) [14,16,27,28]. All other mutations are present in <5% of cases [16]. Notably, some studies clearly indicated that some genetic alterations may be more prevalent in the relapsed forms of MM and different genetically-defined populations [29]. Thus, *BRAF* mutations, including an activating *V600E* mutation, are present in 4–9% MM cases at diagnosis, while relapsed forms may additionally acquire *BRAF* gene mutations (up to 18%) [13,28,30]. Similarly, Xu et al. (2017) showed a significant increase in MAPK pathway mutations in relapsed forms of MM in comparison with primary MM (mainly due to an increase in *NRAS* gene mutations) [31]. A study of Kortum et al. also revealed an increased prevalence of *KRAS*, *NRAS*, and/or *BRAF* gene mutations (72%), as well as mutations of several other genes including *TP53* (26%), *CRBN* (12%), and CRBN pathway genes (10%) [32].

## 3. Current Treatment Strategies for Multiple Myeloma

Despite the improvements in treatment and therapeutic strategies in newly diagnosed MM in the last 2–3 decades, MM remains an incurable disease in the majority of patients. Thus, there is still an unmet need to develop more efficacious treatments. After the start of autologous stem cell transplantation (ASCT) in the early 1980s, no significant developments in the therapy of MM were achieved for many years. Almost two decades later, with the clinical introduction of the first IMiD (thalidomide) [33] and the discovery of the proteasome as a novel potential therapeutic target and its effective targeting treatments, a new era in MM therapy has begun [34]. The approval of bortezomib, the first proteasome inhibitor, was obtained in the USA and in Europe in the early 2000s. The second generation of IMiDs includes lenalidomide, aimed to treat patients with newly diagnosed and relapsed MM. With the approval of multiple new agents over the last 10 years, a change in treatment strategies was observed. As a result, the combination of proteasome inhibitors with immunomodulatory agents has become the therapeutic backbone in the upfront and further treatment lines, often being supplemented with steroids, antibodies, cytostatic drugs, and autologous transplantation in triplet or quadruplet regimes [5,35].

Here we summarize the most commonly used and effective therapeutic strategies in newly-diagnosed and relapsed myeloma.

### 3.1. Proteasome Inhibitors

The proteasome is an enormous multiprotease complex and is physiologically responsible for the degradation of the majority of intracellular proteins. As such, the proteasome plays an essential role in maintaining protein homeostasis and regulates several biological processes, such as signal transduction, cell survival, DNA repair, apoptosis, and antigen presentation [36]. In the last 30 years, the proteasome has been extensively explored as a target for cancer therapy, leading to clinical success in terms of survival of proteasome inhibitors in the treatment of MM. Now, three proteasome inhibitors are widely used in the clinical routine: bortezomib, carfilzomib, and ixazomib. 

#### 3.1.1. Bortezomib

In the beginning of the new millennium, after showing promising results in preclinical models, a phase I trial investigated bortezomib for its safety and tolerability in cancer patients [37]. Bortezomib showed a well-tolerated safety profile, but exhibited some adverse events such as fatigue, fever, thrombocytopenia, and peripheral neuropathy. The SUMMIT phase II trial yielded impressive efficacy in terms of response rate (35%) and duration of response (median 12 months) in heavily pre-treated MM patients [38]. The APEX phase III trial investigated bortezomib vs. dexamethasone in patients progressing on first-line therapy. Bortezomib was superior to cortisone therapy, showing higher response rates (43% vs. 9%) and longer overall survival (29.8 vs. 23.7 months) [39]. This led to the approval of bortezomib in the USA in 2005 in the second-line treatment of MM patients. Three years later, bortezomib received approval in newly diagnosed MM patients in combination with melphalan and prednisone. In the phase III trial VISTA, 682 patients with MM were randomized to melphalan and prednisone, with and without bortezomib. The triple arm yielded impressive data compared to the control group, showing a prolongation of time-to-progression (24.0 vs. 16.6 months, HR 0.48), higher objective response rates (71% vs. 35%), and a longer median duration of response (19.9 vs. 13.1 months) [40]. 

Nowadays, bortezomib is commonly used as a mainstay agent in the first-line setting, in combination with other drugs, such as immunomodulatory agents (thalidomide or lenalidomide), cytotoxic drugs (melphalan), and corticosteroids (dexamethasone or prednisone).

#### 3.1.2. Carfilzomib

Carfilzomib is a representative of the second-generation of proteasome inhibitors. In contrast with bortezomib, carfilzomib shows a higher selectivity to the proteasome, covering more of the proteolytic subunits, and lower off-target activity, causing fewer side effects (lower rates of peripheral neuropathy) [41]. Furthermore, it was observed that a subset of patients who did not respond to initial bortezomib therapy could still benefit from carfilzomib treatment. The phase III ASPIRE trial confirmed this data in providing evidence for effectiveness of carfilzomib-containing regimens against relapsed MM, including patients treated prior with bortezomib [42]. In total, 792 patients were randomly assigned to carfilzomib in combination with lenalidomide/dexamethasone, or to lenalidomide and dexamethasone alone. The addition of carfilzomib resulted in a longer progression-free survival compared with double-therapy (26.3 vs. 17.6 months, HR 0.69), with a similar toxicity grade profile (grade 3 or higher: 83.7% vs. 80.7%) and a higher quality of life. Only recently, the overall survival data were published. The effectiveness of the carfilzomib arm was confirmed by prolonging the overall survival by approximately 8 months (48.3 vs. 40.4 months, HR 0.79) [43].

ENDEAVOR is another head-to-head phase III trial comparing carfilzomib with bortezomib, both combined with dexamethasone, in patients with RRMM. Already in the interim analysis, a superiority of carfilzomib over bortezomib in terms of extending overall survival was shown (47.6 vs. 40.0 months, HR 0.79). Furthermore, carfilzomib plus dexamethasone showed a better safety profile, especially with regard to peripheral neurotoxicity. However, more cardiovascular events were detected in the carfilzomib group [44].

Those two phase III trials have led to the approval of carfilzomib as a standard therapy for RRMM, and Carfilzomib may become a part of front-line therapy in the near future.

#### 3.1.3. Ixazomib

Bortezomib and carfilzomib are administered intravenously or subcutaneously, respectively, while an orally available drug was missing until the development of ixazomib. In 2015, ixazomib received an FDA approval as the first orally bioavailable proteasome inhibitor. Ixazomib, in combination with lenalidomide plus dexamethasone, showed positive results in several phase I-III clinical trials [45]. In the placebo-controlled phase III TOURMALINE-MM1 trial, ixazomib was tested in combination with dexamethasone and lenalidomide in RRMM patients [46]. A significant prolongation of progression-free survival (primary endpoint) was observed for the experimental arm compared to the control arm (20.6 vs. 14.7 months, HR 0.74). Adverse events and quality of life were comparable between the two groups.

Although positive signals have come from different clinical trials, head-to-head comparisons with bortezomib or carfilzomib are lacking. However, clinical trials are ongoing (clinicaltrials.gov, i.e., NCT 03416374) and will hopefully show if ixazomib can be used in similar therapeutic strategies as bortezomib or carfilzomib. Furthermore, the oral bioavailability and long half-live predestines Ixazomib for maintenance and long-time treatment strategies. 

The huge success of proteasome inhibitors changed the therapeutic strategies and influenced the survival of MM patients dramatically. As a result, currently, three new-generation proteasome inhibitors (oprozomib, delanzomib, marizomib) are in the clinical pipeline with the aim of enhancing clinical efficacy and outcome. 

### 3.2. Immunomodulatory Drugs (IMiDs)

The introduction of immunomodulatory drugs (IMiDs), further improved long-term survival of MM patients. Anti-myeloma activity of IMiDs is mediated by cytokine modulation and immunomodulatory, anti-angiogenic, anti-inflammatory, as well as cytotoxic effects. The IMiDs bind to cereblon, their direct protein target, and regulate the ubiquitination of key transcription factors [47]. Due to their lower toxicity and higher anti-myeloma potency, two analogs of the first IMiD thalidomide, lenalidomide, and pomalidomide, were approved for MM treatment settings from first-line to maintenance [48,49,50]. Lenalidomide is widely used in all phases in MM treatment, encompassing newly-diagnosed and relapsed/refractory patients with impressive response rates. The full potential of IMiD-based therapeutic approaches can be exploited in combination therapies with PIs, steroids and/or monoclonal antibodies [11]. 

### 3.3. Monoclonal Antibodies (MoAbs)

Furthermore, introduction of the first monoclonal antibodies into multiple myeloma therapy started a new era in MM treatment. Daratumumab, targeting CD38 as a highly and constantly expressed MM cell surface protein, is the first antibody that was approved by the FDA for the treatment of both relapsed/refractory [51] and newly-diagnosed MM patients [52]. Regimens comprised daratumumab monotherapy, as well as both PI- and IMiD-based combination therapies.

Elotuzumab, directed against human MM cell surface glycoprotein receptor CS1/SLAMF7, has been approved for treatment of RRMM patients [53] in combination with LenDEx, while showing only minor activity in monotherapy [54]. Multiple other elotuzumab, daratumumab, or other MoAb-based combinations are under investigation. Due to the relatively low toxicity of antibody-based therapies, this may comprise long-term and maintenance treatment strategies in the future [54,55].

Monoclonal antibodies show distinct mechanisms of action, inducing multiple myeloma cell death involving the use of immune cells or complement proteins, or via acting on the host’s immune system independently. The addition of monoclonal antibodies to the MM therapeutic landscape is expected to enable more effective targeted treatment of myeloma, resulting in improved outcomes with a favorable side effects profile.

### 3.4. Histone-Deacetylase (HDAC) Inhibitors

A variety of epigenetic alterations along with genetic mutations is essential for cancer initiation and progression. Modifying histones by acetylation is, along with DNA methylation, an option to alter gene expression, and is controlled by two enzymes: histone-deacetylases (HDAC) and histone-acetyltransferases. Upregulation of HDAC leads to proliferation and migration of cancer cells by altering DNA repair genes and anti-oncogenes [56,57]. Thus, inhibiting HDAC is a potential anti-cancer target. 

The first HDAC inhibitor tested in MM was vorinostat, an oral pan-HDAC inhibitor. After positive signals coming from preclinical and clinical phase I/II trials in combinations with other agents (but not as monotherapy), the phase III VANTAGE-088 was initiated. In this randomized study, vorinostat was tested with bortezomib against bortezomib and placebo. Although the obtained results were statistically significant, the trial showed only a marginal prolongation of progression-free survival (7.6 months vs. 6.8 months, HR 0.77), resulting in a lack of clinical relevance [58]. 

On the other hand, panobinostat, another oral pan-HDAC inhibitor, obtained approval for the treatment of relapsed MM patients. In a phase III trial, panobinostat was tested in a similar setting to vorinostat in combination with bortezomib and dexamethasone, compared to bortezomib and dexamethasone combined with placebo. However, in contrast to the results from the VANTAGE-088 study, this clinical trial (PANORAMA-1) showed a significant prolongation of progression-free survival of approximately 3 months (11.99 months vs. 8.08 months, *p* < 0.0001) [59]. However, the overall survival data were modest, with 40.3 months for the panobinostat arm and 35.8 months for the control arm (HR 0.94) [60]. Interestingly, panobinostat showed higher activity in double-refractory, heavily pre-treated patients than in a second line setting.

Because of the higher adverse events rates, especially diarrhea and arrhythmias, the role of panobinostat in the clinical routine remains unclear. Modifications in the dosing and application of panobinostat, and the agents used in combination, may reduce toxicity, as the second phase of the PANORAMA-1 trial could show [61]. The phase II PANORAMA-3 trial is currently ongoing and analyzes the safety and efficacy of three different dose-regimens in combination with subcutaneous bortezomib and oral dexamethasone (clinicaltrials.gov; NCT02654990). The results of this study are expected in 2019. 

Panobinostat was also tested with other combinations in different clinical phase I/II trials. In a multicenter, open-label phase I/II trial (MUK-six), panobinostat in combination with bortezomib, thalidomide and dexamethasone yielded an impressive objective response rate of 91%, and was well-tolerated [62]. On the other hand, a phase I/II study investigated the efficacy and safety of the combination of panobinostat and carfilzomib. An objective response rate of 67% and a progression-free survival of 7.7 months were achieved [63].

Some preclinical data indicate that specific HDAC inhibitors, instead of the use of pan-HDAC inhibitors, could be more active and less toxic. In particular, targeting HDAC6 was shown to be attractive in myeloma cells [64]. Ricolinostat (ACY-1215) showed encouraging efficacy, with a better toxicity profile than panobinostat [65].

The use of HDAC inhibitors is an additional weapon against RRMM. However, the toxic profile of panobinostat must be kept in mind when treating patients. Ongoing trials should establish the right dose of panobinostat and the effectiveness of the selective HDAC6 inhibitor ricolinostat. 

## 4. Experimental Therapies: Immune Checkpoint (PD-1/PD-L1) Inhibitors in Multiple Myeloma

Checkpoint receptors play a key role in maintaining immune tolerance in the human body. These include inhibitory molecules (e.g., programmed death 1 (PD-1) and cytotoxic T lymphocyte antigen 4 (CTLA-4)) and stimulatory molecules (e.g., CD28) on T-cells and PD-L1 on cancer cells. 

Numerous preclinical and clinical studies have confirmed that upregulation of PD-1 and its ligand PD-L1 (as well as PD-L2) may promote tumor growth, while inhibiting this mechanism through anti-PD-1/PD-L1 drugs may enhance antitumor immune responses [66]. Most remarkable therapeutic results have been shown in non-small cell lung carcinoma, malignant melanoma, advanced urothelial carcinoma, metastatic renal cell carcinoma, Merkel cell carcinoma, classical Hodgkin lymphoma, advanced gastric/gastroesophageal junction (GEJ) adenocarcinomas, and other MSI-H/mismatch repair deficient (dMMR) solid cancers [67,68,69,70,71,72,73,74,75,76,77,78]. 

PD-L1 expression has been described in myeloma cells [79]. Moreover, Tamura et al. showed that the bone marrow microenvironment may upregulate PD-L1 expression on myeloma cells, contributing to both T-cell downregulation and enhancing aggressiveness of myeloma cells [80]. Several studies confirmed that PD-L1 expression in MM directly correlates with the progression of the disease, and is strongly associated with relapsed/refractory forms of MM [9,80,81,82,83]. 

Clinical studies that assessed the benefit of monotherapy approaches with immune checkpoint inhibitors in patients with MM reported unsatisfactory therapeutic benefits of anti-PD-1/PD-L1 drugs [7,84]. A recent study of Lesokhin et al. revealed that only 2/27 (8%) RRMM patients had a complete and/or partial response following the treatment with nivolumab (anti-PD-1 monoclonal antibody) [85]. In contrast, more than 50% of patients with diffuse large B-cell lymphoma and follicular lymphoma exhibited a complete and/or objective response to the immune checkpoint inhibitor [85]. While the interim results of two clinical trials investigating IMID/anti-PD1 combinations reported positive results with regards to progress-free survival (PFS) with pembrolizumab-pomalidomide and dexamethasone (KEYNOTE-183), as well as pembrolizumab-lenalidomide and dexamethasone (KEYNOTE-185) in heavily pre-treated patients, these trials [86,87] could not confirm better response rates and PFS on further follow-up. Moreover, IMiD checkpoint antibody combinations trials were only recently stopped by the FDA because of an excess in mortality (data presented at the ASCO 2018 meeting) [88,89]. Taken together, basic experimental and clinical studies clearly indicate that immune checkpoint inhibitors alone are not sufficient to achieve a therapeutic benefit in MM patients, and are associated with a severe mortality in IMiD-based combinations. The focus of research in immune-oncology in myeloma therefore has changed to other fields (e.g., myeloma specific CAR-T cells) [90].

## 5. Treatment of Relapsed/Refractory Myeloma and the Role of Molecular Profiling

The genetic heterogeneity of MM, known to increase over multiple relapses and treatment lines [91], together with subclonal dominance changes, favors the choice for combination treatment in RRMM whenever clinically feasible. Other factors that should be taken into account when deciding how to sequence and combine the panoply of new and old drugs described in the section of current treatment options are patient and disease characteristics, as well as the biological risk status. This comprises, among other factors, patient’s age, performance and frailty status, co-morbidities, and personal preferences on one side, and duration and depth of the clinical response to previous treatment lines, as well as anamnestic and/or ongoing side effects and toxicities on the other.

With rare exceptions for some targeted drugs (e.g., BRAF inhibitors) [92], all of this is based purely on clinical reasoning, while clear-cut predictive markers to tailor a rationale therapy allocation are lacking. Given the endless array of possible permutations of myeloma drugs, this constitutes a major unmet medical need. Furthermore, nearly all MM patients will reach the point of being refractory to all registered treatments.

The efficacy of treatment according to a molecular profile, which is usually performed on tumor tissue, is currently evaluated in different solid tumors worldwide [93]. The scope of molecular profiling is to find a potential predictive target using different analysis methods, such as next-generation sequencing (NGS) and/or immunohistochemistry (IHC), and to finally treat the patients accordingly with a targeted therapy. This approach is widely used and gives the possibility to treat patients with relapsed and refractory cancers in a personalized manner. 

## 6. The Role of Molecular Profiling in Multiple Myeloma: Authors’ Initial Experiences Based on Multiplatform Molecular Profiling

In hematological diseases, molecular profiling is in an early stage of development. While hematologists started to use targeted therapy in the model disease context of CML decades ago, the realization that dominant driver-dependent malignancies are more the exception than the rule took some time to settle in hematologists’ minds, while solid tumors were understood to be genetically complex diseases quite earlier. 

MM is also known to be without universal driver mutations (see paragraph on the genomic profile of MM), although multiple common genetic alterations have been described. Furthermore, predictive markers of pharmacotherapy resistance or sensitivity have been published but only rarely tested in a prospective fashion (e.g., *FLT3*, *NEK2*, *EZH2*, *GRP78*, *PSMB5*, and *CRBN*) [31,94,95,96,97,98,99,100].

By applying a comprehensive profiling to myeloma samples, not only myeloma specific alterations, but also genetic alterations or protein expression alterations usually not considered in MM can be frequently identified. 

**Figure 1:**
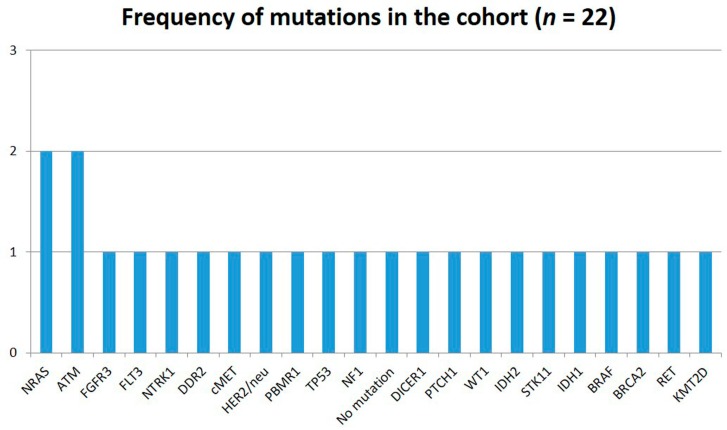
Mutational profile (frequency of mutations) of 22 samples from advanced myeloma patients (all beyond third line settings) using a next-generation sequencing (NGS) assay.

In our pilot series (within the ONCO-T-PROFILE project) [101], we analyzed bone marrow samples from 22 advanced myeloma patients (all beyond the third line settings) using a multiplatform approach: immunohistochemistry (IHC), fluorescent in-situ hybridization (FISH) and next-generation sequencing (NGS) (592 genes, NextSeq, Illumina, San Diego, CA, USA). All assays were performed in a CLIA/CAP/ISO15189 certified clinical laboratory (Caris Life Sciences, Phoenix, AZ, USA), as previously reported [102,103,104]. We were able to identify a broad spectrum of mutations. In line with the complex genetic structure of MM, most of the mutations were detected only in single patient samples (e.g., *NRAS*, *ATM*, *NTRK1*, *cMET*, *HER2, BRAF*, *IDH1*, *IDH2*) (see Figure 1), and most of them were not targetable in MM and other cancers beyond clinical trials. 

**Figure 2:**
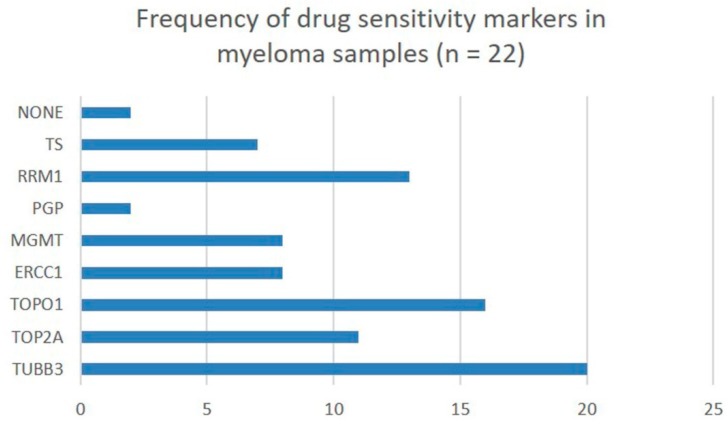
Various drug sensitivity biomarkers explored by immunohistochemistry in a cohort of 22 multiple myeloma patients. TS = thymidylate Synthase; RRM1 = tibonucleotide reductase M1; PGP = P-glycoprotein 1; MGMT1 = *O*^6^-alkylguanine DNA alkyltransferase; ERCC1 = excision repair cross-complementing group 1; TOPO1 = topoisomerase I; TOP2A = topoisomerase IIα; TUBB3 = class III β-tubulin.

**Figure 3:**
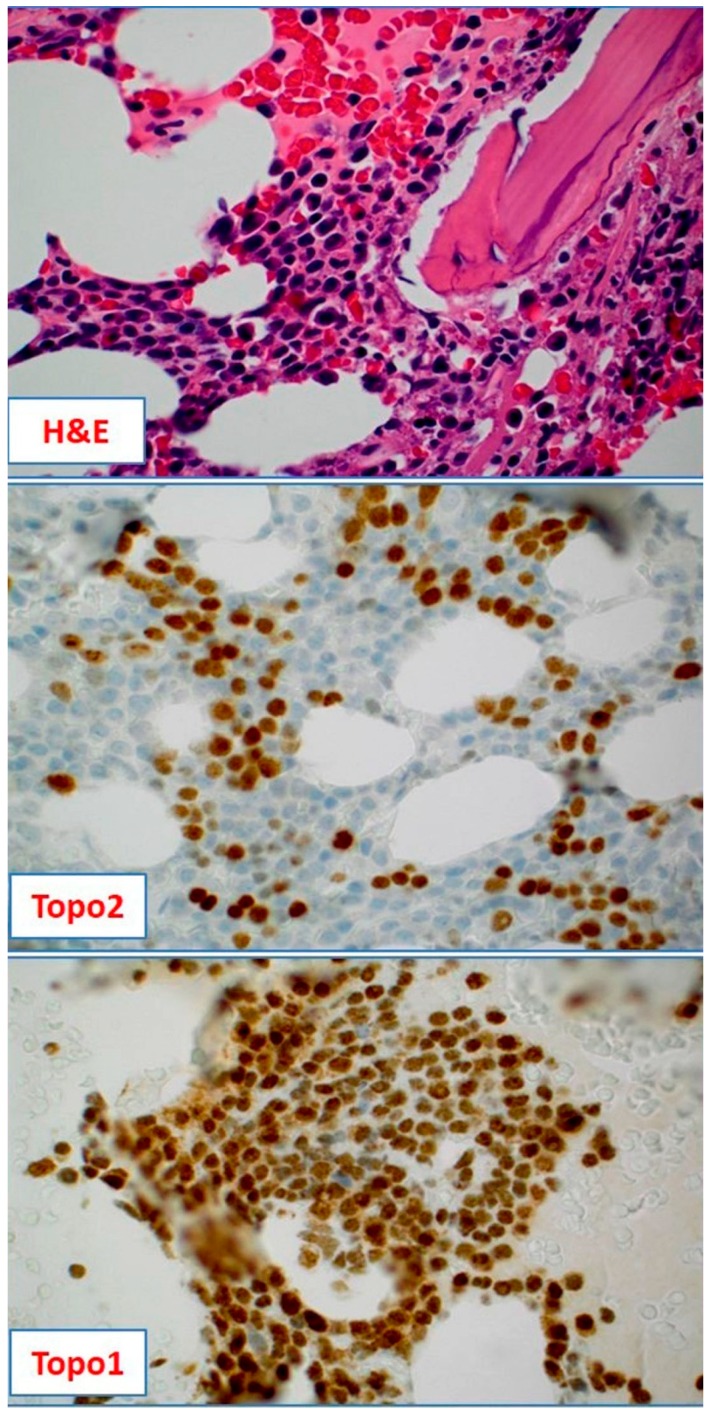
Hematoxylin-Eosin (H&E) staining of a bone marrow biopsy from a relapsed and heavily pretreated multiple myeloma patient revealing a diffuse infiltration with neoplastic myeloma cells (10× magnification); the tumor cells were positive for Topo2 (40% of the cells positive) and Topo1 (90% of the cells positive) (20× magnification).

With respect to conventional chemotherapy sensitivity biomarkers, a broad array was identified in all but two samples (shown in Figure 2). This includes biomarkers that predict sensitivity to gemcitabine (Ribonucleotide reductase M1 or RRM1), taxanes (Class III β-tubulin or TUBB3), anthracyclines (Topoisomerase IIα or Topo2/TOP2A), topotecan (Topoisomerase I or Topo1/TOPO1), thymidylate synthase inhibitors (Thymidylate Synthase), temozolamide (*O*^6^-alkylguanine DNA alkyltransferase or MGMT), platinum (Excision repair cross-complementing group 1 or ERCC1), and multidrug resistance biomarker against vinblastine, doxorubicine, and tyrosine kinase inhibitors (P-glycoprotein 1 or PGP) (Figure 2 and Figure 3).

Up to now, three patients received four lines of treatment based on profiling results in far advanced disease settings (previous lines of therapy 6–12). Although the numbers are still small, the results were quite encouraging, with PFS intervals superior to the respective patient’s previous lines in three out of the four cases (one is ongoing). Most impressive, a PFS of nine months with an anthracycline-based therapy in a ninth line setting was achieved in one patient. The treatment was based on a strong Topo2 protein expression (Figure 3); the same patient received a palliative chemotherapy with topotecan in 12th line later on (based on Topo1 expression) (Figure 3).

Our clinical experience provided here is limited by the small number of patients (*n* = 3) that were treated following profiling results described above. Therefore, we plan to extend our research and to compare results of treatments applied in the future based on profiling results with the last treatment they had beforehand. This is considered a standard approach in therapeutic profiling studies [101,105].

## 7. Conclusions

Multiple myeloma is a genetically complex disease on both the chromosomal and subchromosomal levels, with no unique dominant driver mutations. Despite multiple treatment options and significantly prolonged survival [106], MM remains incurable in the vast majority of patients. Although very intensive treatment approaches may be curative in some MM patients [107], this approach has been challenged by other investigators [108]. Thus, standard therapies will eventually be of no use in all of these patients. As our results show a pattern of diverse, rare driver mutations and frequent chemotherapy sensitive markers, a “targeted revival” of old-fashioned cytotoxic therapeutics may be used in rational combination with typical MM drugs. 

To personalize tailored treatments for patients with relapsed/or refractory MM, the mutational and expression status of drug sensitivity and insensitivity biomarkers appears to be the most rational approach to use. Setting up such a program, we hope to open a new avenue for more effective, personalized treatment approaches for a highly complex disease that has always defied one-size-fits-all solutions.
